# Evaluation and assessment of clique arrangements for the estimation of omnipolar electrograms in high density electrode arrays: an experimental animal model study

**DOI:** 10.1007/s13246-023-01287-8

**Published:** 2023-06-26

**Authors:** Samuel Ruipérez-Campillo, Marina Crespo, Álvaro Tormos, Antonio Guill, Antonio Cebrián, Antonio Alberola, Jakob Heimer, Francisco J. Chorro, José Millet, Francisco Castells

**Affiliations:** 1 ITACA Institute, Universitat Politècnica de València, Valencia, Spain; 2 Department of Information Technology and Electrical Engineering, Swiss Federal Institute of Technology (ETH), Zürich, Switzerland; 3 School of Medicine, Stanford University, Stanford, CA USA; 4 Departamento de Fisiología, Universitat de València, Valencia, Spain; 5 Centro de Investigación Biomédica en Red Enfermedades Cardiovascular (CIBERCV), Madrid, Spain; 6 Department of Mathematics, Seminar for Statistics, Swiss Federal Institute of Technology (ETH), Zürich, Switzerland; 7 Departamento de Medicina, Universitat de València, Valencia, Spain; 8 Servicio de Cardiología, Hospital Clínic Universitari de València, Valencia, Spain

**Keywords:** Omnipolar electrograms, High density electrode arrays, Experimental animal study, Local substrate exploration, Signal processing, Cardiac catheters

## Abstract

High-density catheters combined with Orientation Independent Sensing (OIS) methods have emerged as a groundbreaking technology for cardiac substrate characterisation. In this study, we aim to assess the arrangements and constraints to reliably estimate the so-called omnipolar electrogram (oEGM). Performance was evaluated using an experimental animal model. Thirty-eight recordings from nine retrospective experiments on isolated perfused rabbit hearts with an epicardial HD multielectrode were used. We estimated oEGMs according to the classic triangular clique (4 possible orientations) and a novel cross-orientation clique arrangement. Furthermore, we tested the effects of interelectrode spacing from 1 to 4 mm. Performance was evaluated by means of several parameters that measured amplitude rejection ratios, electric field loop area, activation pulse width and morphology distortion. Most reliable oEGM estimations were obtained with cross-configurations and interelectrode spacings $\le 2$ mm. Estimations from triangular cliques resulted in wider electric field loops and unreliable detection of the direction of the propagation wavefront. Moreover, increasing interelectrode distance resulted in increased pulse width and morphology distortion. The results prove that current oEGM estimation techniques are insufficiently accurate. This study opens a new standpoint for the design of new-generation HD catheters and mapping software.

**Supplementary Information:**

The online version contains supplementary material available at 10.1007/s13246-023-01287-8.

## Introduction

Local examination of the cardiac tissue is crucial for the characterisation of the electrophysiologic substrate [1], found to be key to better understand the mechanisms that trigger and sustain cardiac arrhythmias such as atrial fibrillation [2], atrial tachycardia [3], ventricular tachyarrhythmias [4, 5] and other arrhythmias [6]. Arrhythmogenic substrates usually involve fibrotic regions with anomalous conduction that cause meandering and inhomogeneous routes of the electrical activation [7, 8]. Accurate electrophysiological mapping is then required to identify sites responsible for the arrhythmia, and hence pinpoint candidates for ablation procedures [9, 10].

In order to accurately characterise the electrophysiological substrate, catheters with high-density (HD) arrays of equispaced electrodes are gaining great interest in the field [11]. These electrodes are able to provide an HD activation map of the local tissue, and hence are appropriate to estimate conduction velocity and other features related to inhomogeneities in the propagation of the electrical wavefront [12, 13]. In fact, they have already been introduced to the clinical practice involving successful substrate exploration for the detection of atrial and ventricular disorders [14–16].

Such multielectrode arrays are also referred to as omnipolar electrodes, due to their capability to derive an omnipolar EGM (oEGM), which is a virtual representation of the bipolar EGM (bEGM), recreating the hypothetical signal obtained from a pair of electrodes arranged in the direction of wavefront propagation. The interest of this operation mode is to overcome the sensitivity of bEGMs to the orientation of the electrode pair with respect to the wavefront [17]. Due to this limitation of bEGMs, the low-amplitude and fragmented activations recorded in the case of wavefronts arriving orthogonally to the electrode pair [18, 19] could lead to misinterpretations, such as mistakenly assuming impaired tissue to be the cause of an abnormal signal [20, 21].

Although omnipolar electrodes are claimed to provide an orientation-independent oEGM [11, 22, 23], some orientation dependencies have been reported [24, 25], which may lead to pitfalls in oEGM estimation at some incidence angles. To overcome this limitation, two alternative methods for oEGM estimation have been recently proposed. The first one consists of a prior alignment of the bEGM pair to minimise delay between activations [24]. Yet, ex-vivo animal experiments are a way, according to the authors, to establish if the simulation-proved superior performance of their modified omnipolar EGM translates to clinical counterpart of their simulation-based study [24]. The second one refers to a cross-orientation method by choosing the diagonal bEGMs of the square clique, as opposed to the conventional triangular configuration. This method is proposed as a way to impose coincident bipole centres [25], and hence avoid delays between bEGMs. Although the latter method showed promising results towards overcoming the aforementioned limitations and presenting a more robust approach against propagation angle, this was only tested with simulations based on ideal propagation wavefronts presented as homogeneous and plane waves, which differ from the non-ideal propagation patterns intrinsic to the electrophysiological environment [11]. Thus, there was not enough evidence that this method could work well in a realistic scenario, considering the complexities of cardiac electrophysiology. Therefore, this technique requires further validation in more realistic settings.

Animal models are widely used to test and validate techniques in real biological scenarios [26]. Among them, the isolated perfused heart according to the Langendorff technique is a broadly used ex-vivo model in cardiac electrophysiological research [27].

**Fig. 1:**
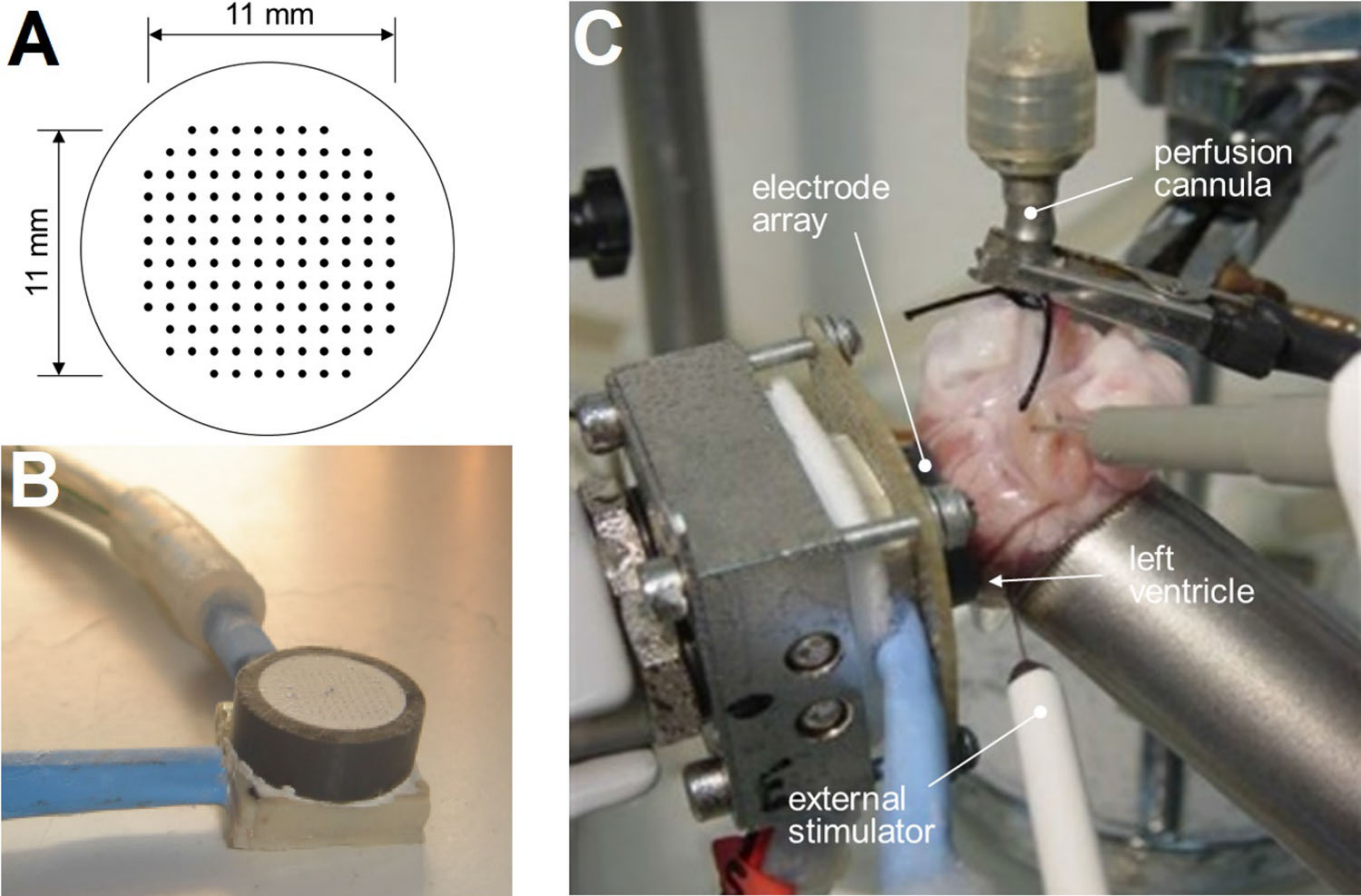
**A** Arrangement of the multielectrode array. **B** Picture of the self-manufactured multielectrode. **C** Experimental setting

In this paper, we present a study on the performance of the cross-orientation method for oEGM estimation using experimental data. For this, a retrospective dataset of isolated perfused rabbit hearts was employed. These experiments have many conditioning factors derived from the physiological environment where this methodology is meant to be used, including recordings of real epicardial activations, variability among the samples or electrode limitations in a clinical environment, among others. The signals were obtained with an HD multielectrode array containing 128 unipolar electrodes with 1 mm spacing. Objective features such as activation amplitude, pulse duration and loop width are analysed. Furthermore, the effects of interelectrode distance can be assessed given the configuration of the electrode array designed for this experiment (Fig. 1A).

## Materials

Thirty-eight recordings from 9 retrospective experiments performed on isolated perfused rabbit hearts according to the Langendorff technique were used [28]. Two recordings per heart were used, stimulated at 4 and 6 Hz, and three series were selected per recording. Those with lower quality, noise, or artifacts were discarded. In each experiment, a self-manufactured multielectrode consisting of 128 stainless steel electrodes (interelectrode distance 1 mm; diameter 0.125 mm) [29] was positioned on the epicardial surface of the anterior wall of the left ventricle. A bipolar epicardial stimulating electrode was used (diameter, 0.125 mm; interelectrode distance, 1 mm), always positioned at the same location, proximally to the external lateral side of the recording electrode (Fig. 1) and connected to a GRASS S88 stimulator equipped with a stimulus isolation unit. Signals from several series (epicardial temperature 37 $^{\circ }$C) with ventricular pacing (4 Hz and 6 Hz) were used for this research. Stimuli were applied via a train of 2 ms pulses with voltage of twice the diastolic threshold. Electrogram recordings were obtained through a cardiac electrical activity mapping system (MAPTECH; Waalre, The Netherlands). The reference electrode consisted of a $4 \times 6$ mm silver plate located over the cannulated aorta. All signals were amplified with a gain of 100–300, bandwidth filtered (1 Hz-400 Hz), multiplexed, and digitised (resolution, 12 bits). The sampling rate was 1000 Hz per channel. Experiments were performed at the Laboratory of Experimental Cardiac Electrophysiology at the Department of Physiology of the University of València, Valencia, Spain. The protocol for the experiments was previously approved by the University of València Local Committee

## Methods

### Clique configurations

**Fig. 2:**
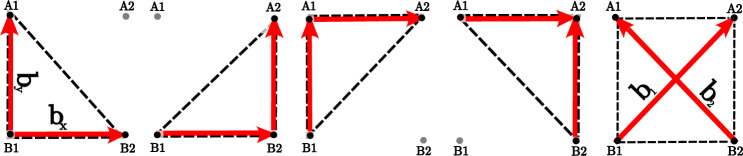
Configurations of a clique of electrodes , , , , and

Considering the electrode location as the pair (*i*, *j*), where *i* and *j* are alphabetic and numeric ordinal indices, respectively, the unipolar EGM (uEGM) in a particular electrode will be referred to as $u_{ij}(t)$ (e.g. $u_{\textrm{A}2}(t)$). From the subtraction of unipole pairs, it is possible to derive a bipolar EGM bEGMs). From a cubicle arranging $2\times 2$ electrodes, a clique is defined as a pair of orthogonal bEGMs, as depicted in Fig. 2. Depending on the bEGM arrangement, several configurations can be considered, such as triangular (with 4 different orientations: , ,  and ) or cross cliques. For the sake of clarity, let us denote different clique configurations as , , ,  and , respectively. Considering a $2\times 2$ cell with electrodes A1, A2, B1 and B2, bipole pairs for each different clique configuration are defined as follows (see Fig. 2):

- For a lower left triangular clique :
  $$\begin{aligned} b_x(t) = u_{\textrm{B}2}(t) - u_{\textrm{B}1}(t) \\ b_y(t) = u_{\textrm{A}1}(t) -u_{\textrm{B}1}(t) \end{aligned}$$
- For a lower right triangular clique :
  $$\begin{aligned} b_x(t) = u_{\textrm{B}2}(t) - u_{\textrm{B}1}(t) \\ b_y(t) = u_{\textrm{A}2}(t)-u_{\textrm{B}2}(t) \end{aligned}$$
- For an upper left triangular clique :
  $$\begin{aligned} b_x(t) = u_{\textrm{A}2}(t) - u_{\textrm{A}1}(t) \\ b_y(t) = u_{\textrm{A}1}(t)-u_{\textrm{B}1}(t) \end{aligned}$$
- For an upper right triangular clique :
  $$\begin{aligned} b_x(t) = u_{\textrm{A}2}(t) - u_{\textrm{A}1}(t) \\ b_y(t) = u_{\textrm{A}2}(t)-u_{\textrm{B}2}(t) \end{aligned}$$
- For a cross clique :
  $$\begin{aligned} b_1(t) = u_{\textrm{A}2}(t) - u_{\textrm{B}1}(t)\\ b_2(t) = u_{\textrm{A}1}(t)-u_{\textrm{B}2}(t) \end{aligned}$$

Notice that for the cross clique , a correction to align the coordinate system with the bipole orientation, and hence retrieve $b_x(t)$ and $b_y(t)$, is required. That alignment is achieved by means of a counterclockwise $\frac{\pi }{4}$ *rad* rotation:

1 $$\begin{aligned} {\textbf{b}}(t) = \begin{bmatrix} \cos (\frac{\pi }{4}) &{} -\sin (\frac{\pi }{4}) \\ \sin (\frac{\pi }{4}) &{} \cos (\frac{\pi }{4}) \end{bmatrix} \cdot \begin{bmatrix} b_{1}(t) \\ b_{2}(t) \end{bmatrix}, \end{aligned}$$

where ${\textbf{b}}(t) = [b_x(t) \qquad b_y(t)]^\textrm{T}$ is the Cartesian bipole pair, which applies regardless of the clique configuration.

### oEGM estimation

The oEGM is defined as a virtual bEGM reproducing a hypothetical recording by an electrode pair oriented in the direction of the wavefront propagation. Although the oEGM cannot be directly measured from the multielectrode grid, it can be estimated from a mathematical transformation of the bipole pair ${\textbf{b}}(t)$. From its orthogonal components, the electric field generated by the cardiac electrical activation can be represented as a loop pointing in the direction of propagation. Under the conditions of planar and homogeneous wavefronts (more likely to apply in small-size cliques), it can be stated that the narrower the loop, the more precise the description of the electric field [24, 25].

The omnipole *o*(*t*) can be estimated from the projection of ${\textbf{b}}(t)$ along the direction of the wavefront propagation. Ideally, this transformation yields a signal exhibiting an activation with maximal amplitude. Equally, a projection onto a perpendicular axis provides a residual signal *r*(*t*) with low amplitude. In this line of thinking, the oEGM is computed from the projection that maximises the ratio of the omnipolar peak amplitude to the peak amplitude of the residual. With $\Psi _{\textrm{w}}$ denoting the direction of the wavefront, a rotation angle that maximises the ratio between the amplitude peak of the projected signal and the peak of the orthogonal projection can be computed by solving the following optimisation problem:

2 $$\begin{aligned} \theta _{\textrm{o}} = \underset{\theta }{{\text {argmax}}}\left[ \frac{\max \left( \left[ \cos \theta \hspace{5.0pt}-\sin \theta \right] {\textbf{b}}(t)\right) }{\max \left| \left[ \sin \theta \hspace{5.0pt}\cos \theta \right] {\textbf{b}}(t)\right| }\right] , \end{aligned}$$

where $\theta _{\textrm{o}}$ is the angle that retrieves the projection yielding an estimation of the oEGM ${\hat{o}}(t)$:

3 $$\begin{aligned} \begin{bmatrix} {\hat{o}}(t) \\ r(t) \end{bmatrix} = \begin{bmatrix} \cos (\theta _{\textrm{o}}) &{} -\sin (\theta _{\textrm{o}}) \\ sin(\theta _{\textrm{o}}) &{} \cos (\theta _{\textrm{o}}) \end{bmatrix} \cdot {\textbf{b}}(t) \end{aligned}$$

As shown in Eq. 3, the residual signal *r*(*t*) is naturally derived as well.

### Assessment of oEGM reliability

As suggested from previous simulations [25], the reliability of oEGM estimations depends on several factors. Some of them are inherent to physiological properties, such as conduction velocity and the morphology of the unipolar signal. In addition, the orientation of the multielectrode with respect to the propagation wavefront also plays a role. All these factors are extrinsic to the technique for oEGM estimation as described above. Furthermore, depending on the clique configuration and interelectrode distance, different versions of the estimated omnipole ${\hat{o}}(t)$ can be obtained. As long as results are not coincident, it can be inferred that a retrieved oEMG cannot be considered the true omnipole but rather an approximation. Therefore, assessing and understanding the limitations of technical issues involved in oEGM reconstruction arises as a key factor when proposing and using reliable settings.

**Fig. 3:**
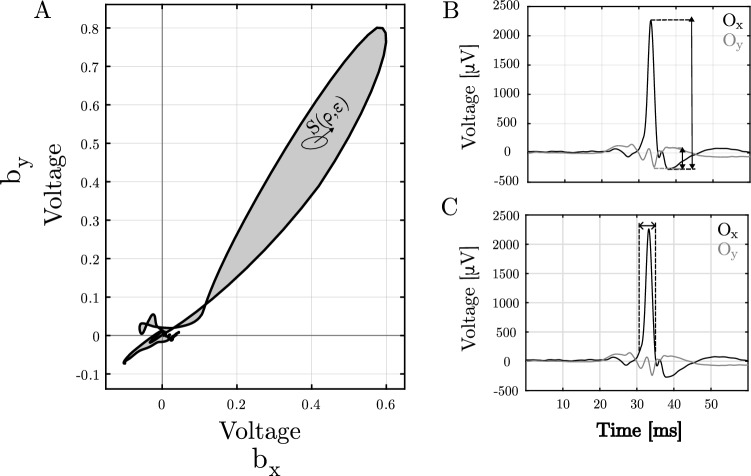
Graphical description of some of the metrics assessed: **A** Normalised loop areas (NLA); **B** Omnipolar ratio (ORR); **C** Pulse width (PW)

Several measurements to assess the reliability of oEGM estimations are proposed:

- oEGM-to-residuum ratio (ORR): Ratio between peak amplitudes of ${\hat{o}}(t)$ and *r*(*t*) activations:
  4 $$\begin{aligned} \textrm{ORR} = \frac{\max ({\hat{o}}(t))}{\max {|r(t)|}} \end{aligned}$$
  The higher this ratio, the better the oEGM estimation.
- Normalised loop area (NLA): Area of the electric field loop described by normalised bipoles that make up the electrical field loop. The rationale for this parameter is that, assuming a planar wave propagating within a small-sized cell, the electric field loop should reflect a straight line. With this assumption, the thinner the loop, the better the estimation and accordingly, lower NLA values suggest more reliable oEGMs. To compute this parameter, the bipoles $b_x(t)$ and $b_y(t)$ are previously normalised to the peak oEGM amplitude. Such normalisation removes amplitude biases in order to reflect a more representative value of the loop shape. The NLA parameter is defined as the surface constrained by the contour of the bipole loop *L*. Parameterising the spatial coordinates according to $\varrho$ and $\xi$, the equation to solve is the surface integral over the magnitude of the cross product of the partial derivatives of the surface element $s(\varrho ,\xi )$ in the plane $\varrho -\xi$ within the limits defined by the curve *L*:
  5 $$\begin{aligned} \textrm{NLA}= \iint _L \left\| \frac{\partial {\textbf{s}}}{\partial \varrho } \times \frac{\partial {\textbf{s}}}{\partial \xi }\right\| \textrm{d} \varrho \mathrm {~d} \xi \end{aligned}$$
  The surface elements are approximated by using an adaptation of the trapezoidal rule, thus avoiding problematic edge cases of triangulation methods such as silver triangles [30].
- Pulse width (PW): The PW is a measure of the elapsed time between the leading and trailing edges of a single pulse (see Fig. 3). The rationale behind this parameter is also related to that of the morphology distortion, as the subtractions of delayed activations will result in an increasing pulse width. From this perspective, the shorter the PW, the better the oEGM estimation.
- Morphology distortion (MD) of ${\hat{o}}(t)$: The rationale for this is the distortion caused by interelectrode spacing sampling. This may occur when the interelectrode distance is not short enough to consider the bipoles $b_x(t)$ and $b_y(t)$ as if they were obtained from infinitesimally close sites. Instead, there can be a significant delay between the activations, so that the bipoles can be regarded as subtractions of delayed versions of the unipole rather than its gradient. Generalising unipolar activations at any site location within a 2D grid, we could define unipolar EGMs as *u*(*t*, *x*, *y*). While being the omnipole *o*(*t*) the gradient of *u*(*t*, *x*, *y*) in the direction of propagation, and considering identical unipolar waveforms in infinitesimally close sites, we estimate the true oEGM at a given site *o*(*t*, *x*, *y*) as the negative time derivative of the unipole:
  6 $$\begin{aligned} o(t,x,y) = - \frac{\partial }{\partial t} u(t,x,y) \end{aligned}$$
  Particularising at the electrodes of the multielectrode array,
  7 $$\begin{aligned} o_{ij}(t) = - \frac{du_{ij}(t)}{dt} \end{aligned}$$
  As long as there are several unipoles involved within a clique, we estimate a reference oEGM $o_{\textrm{ref}}(t)$ after alignment and average all $o_{ij}(t)$ from the electrodes forming the clique. An additional advantage of this averaging is the reduction of common interference and other noise components. The resulting $o_{\textrm{ref}}(t)$ will be then compared to the estimated ${\hat{o}}(t)$ to assess distortion. After amplitude normalisation, MD is measured from root mean squared error (RMSE) between the normalised ${\hat{o}}(t)$ and $o_{\textrm{ref}}(t)$ signals.

A graphical description of some of these parameters is presented in Fig. 3. For each experiment in the dataset, oEGM estimates ${\hat{o}}(t)$ for different clique configurations (, , ,  and ) and interelectrode distances ranging from 1 to 4 mm were obtained. For all cases, performance of oEGM estimation was assessed by means of the parameters described above (i.e. ORR, NLA, PW and MD).

### Statistical analysis

Results are provided as mean ± standard deviation (SD), or median and interquartile range (IQR), if required. For data exploration, the distribution of the independent variables was evaluated using Kolmogorov-Smirnov and Mann–Whitney–Wilcoxon tests.

Two linear mixed models were fit to the data, with the computed metrics (NLA, ORR, PW and MD) as the dependent variable, and configuration and distance, as well as their interaction (*configuration* $\times$ *distance*), as independent variables. Due to the repeated measures on the same rabbit heart origins, a random intercept for the heart origin was included. In both models, the right-skewed dependent variables were *log* transformed.

The first model was fit to the raw data, including an additional random intercept for the subsamples within each combination of rabbit heart origin, configuration and distance. The second model was fit to the aggregated subsamples. The latter was chosen due to its intrinsic simplicity compared to the former, and the similar values obtained for the statistics.

To evaluate the model assumptions, Gaussianity and homoscedasticity of the residuals were studied (see quantile-quantile plots and the residual diagnostics for hierarchical multi-level regression models (DHARMa) in *Supplementary Material*).

P values of model coefficients were obtained using the Satterthwaite’s degrees of freedom method, applying the package *lmerTest*. We performed a post-hoc pairwise testing with *p* value adjustment for correction of multiple comparisons following the Tukey method. Multiple comparisons between each configuration were performed separately for each distance, as were their corrections. A *p* value of < 0.05 was considered statistically significant throughout. The statistical model and comparison tests were designed and run in RStudio.

## Results

**Fig. 4:**
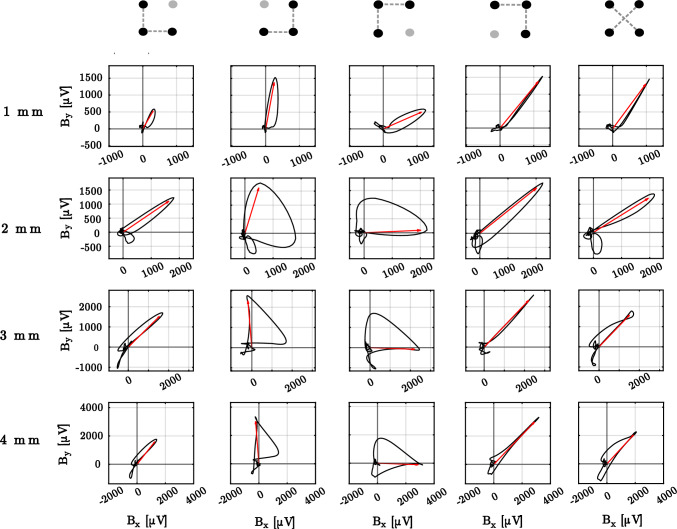
Examples of the bipolar loops (in black) and the corresponding propagation direction (in red) generated by triangular and cross-oriented configurations on the same clique along interelectrode distances 1–4 mm

Figure 4 represents an example of electric field loops created from an experiment and considering different clique configurations and interelectrode distances from 1 to 4 mm. As can be appreciated, different loop patterns were obtained. At closer inspection, the similarity in the morphology of the bEGM loops generated by complementary triangular cliques (for instance  on the one hand and  on the other hand) can be noted. Whereas cliques  and  reconstruct narrower loops pointing at a consistent direction, cliques  and  obtain wider loops with no precise pointing. As a result, triangular configurations  and  fail to accurately detect the direction of propagation. In addition to the triangular cliques, the loop pattern created by the cross clique  is also consistent with the loop patterns of triangles  and , i.e. a narrow loop pointing in the same direction. The reasons for such similarities and differences among patterns will be further discussed in the next section. Moreover, considering the effects of interelectrode distance, it can be observed that with shorter distances, loops become narrower and lower in magnitude.

**Fig. 5:**
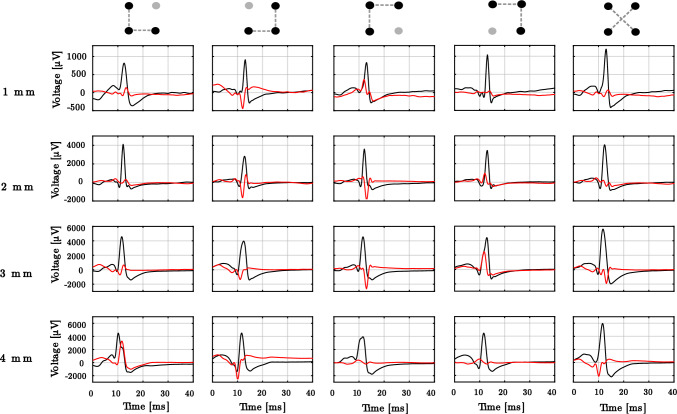
Examples of the omnipoles on the same clique reconstructed by triangular and cross-oriented configurations, with horizontal component of oEGM in black and vertical component in red, along interelectrode distances 1–4 mm

From the bEGMs that make up the electric field loops in Fig. 4, and after the corresponding algebraic rotation according to Eq. 3, the oEGM estimates ${\hat{o}}(t)$ and the orthogonal residual signal *r*(*t*) are computed. These results are depicted in Fig. 5. As can be observed in the 1 mm setting, configurations ,  and  provide estimates with lower amplitude of the residual signal *r*(*t*) (in red). As interelectrode distance increases from 1 to 4 mm, so does the amplitude of the oEGM. Moreover, the residual signal in the direction perpendicular to the wavefront propagation increases to an even larger extent, proportionally to the interelectrode distance. Cliques that provided a wider loop (i.e.  and ) provided a *r*(*t*) signal displaying a significant residue of the electrical activation.

**Table 1:** Results for the different metrics

	{}	{}	{}
1 mm			
NLA	0.130 ± 0.011	0.265 ± 0.032	0.114 ± 0.014
ORR	5.258 ± 0.335	3.722 ± 0.378	5.204 ± 0.339
PW [ms]	5.950 ± 0.441	4.200 ± 0.428	4.324 ± 0.486
MD [$\mu$V]	0.106 ± 0.006	0.086 ± 0.005	0.089 ± 0.006
2 mm			
NLA	0.252 ± 0.026	0.359 ± 0.029	0.161 ± 0.022
ORR	3.695 ± 0.347	2.387 ± 0.128	3.491 ± 0.284
PW [ms]	5.458 ± 0.753	4.618 ± 0.549	5.863 ± 0.493
MD [$\mu$V]	0.105 ± 0.007	0.111 ± 0.008	0.114 ± 0.007
3 mm			
NLA	0.258 ± 0.025	0.308 ± 0.028	0.231 ± 0.033
ORR	3.274 ± 0.226	2.498 ± 0.168	3.133 ± 0.249
PW [ms]	5.378 ± 0.489	5.201 ± 0.538	6.258 ± 0.567
MD [$\mu$V]	0.114 ± 0.006	0.132 ± 0.007	0.133 ± 0.008
4 mm			
NLA	0.284 ± 0.029	0.281 ± 0.019	0.216 ± 0.023
ORR	2.914 ± 0.201	2.745 ± 0.179	2.769 ± 0.148
PW [ms]	6.083 ± 0.380	6.168 ± 0.365	6.732 ± 0.534
MD [$\mu$V]	0.114 ± 0.005	0.136 ± 0.006	0.133 ± 0.008

**Fig. 6:**
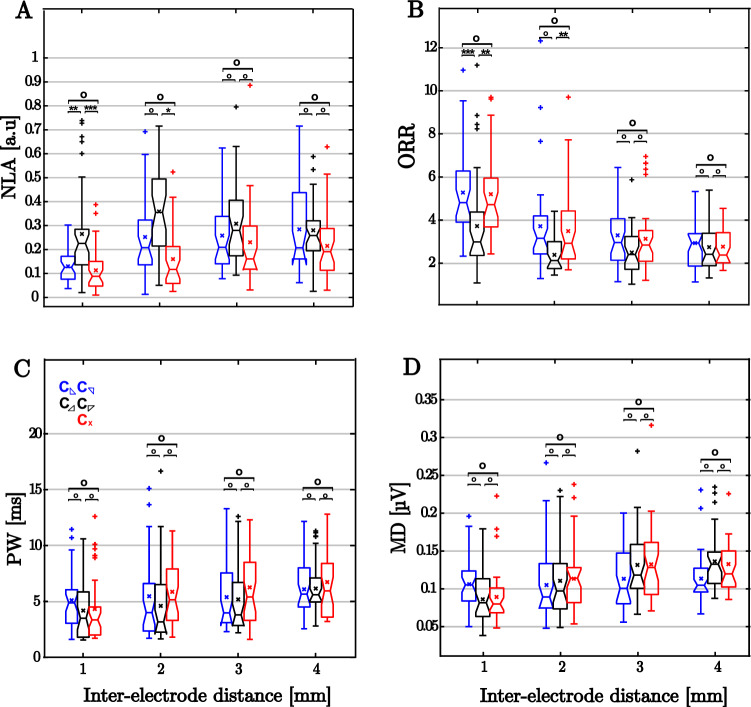
Comparison of the triangular and cross-oriented configurations for the different parameters under study. **A** Normalised loop areas (NLA); **B** Omnipolar ratios (ORR); **C** Morphology distortion of omnipoles (MD); **D** Pulse Width (PW)

Beyond the results of a single experiment illustrated in Figs. 4 and 5, we performed a quantitative analysis by computing parameters ORR, $A_{EFL}$, MD and PW for all the experiments in the dataset. Numerical values are provided in Table 1, and boxplots are depicted in Fig. 6. Subsequently, we carried out the statistical analyses as described in “Statistical analysis” section. The resulting *p* values considering correction for multiple comparisons are given in Table 2.

For close interelectrode spacing (1 mm), the cross configuration  led to equivalent results to the triangular cliques that detected the angle of propagation direction more accurately ( and ), resulting therefore in non-significant *p*-values for amplitude ratio (ORR) and loop area (NLA). However, this similarity was no longer applicable when comparing the results of  to  and . For this comparison, the  clique led to significantly higher OOR and lower NLA values. By increasing interelectrode distance, such differences were reduced, becoming non-significant for spacing $\ge 3$ mm.

**Fig. 7:**
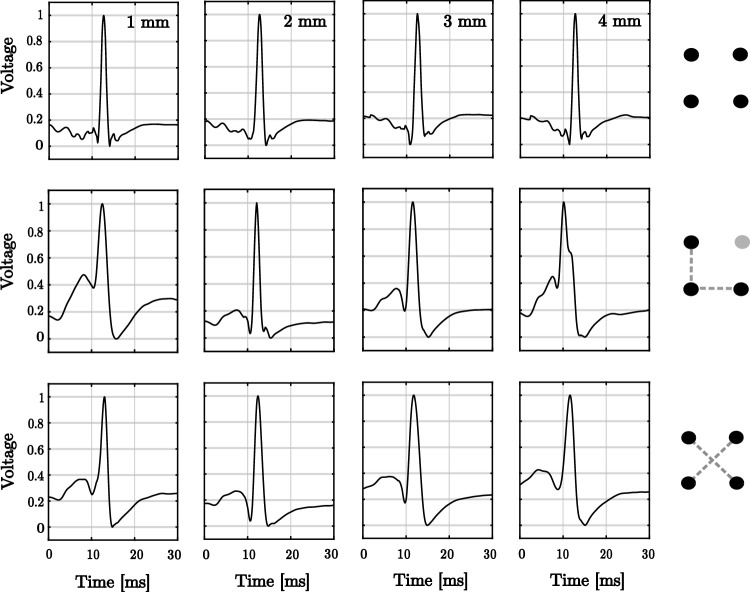
Morphology analysis of oEGMs. Top row: reference oEGM $o_{\textrm{ref}}(t)$ computed from the gradient of the mean uEGM; Central row: estimated oEGM ${\hat{o}}(t)$ for triangular clique ; Bottom row: estimated oEGM for clique . The voltage amplitude has been normalised

Regarding parameters related to pulse morphology PW and MD, comparison among clique configurations provided non-significant results. However, both PW and MD significantly increased with interelectrode distance, especially noticeable for spacing $\ge 3$ mm (see Tables 1 and 3). This effect is reflected in changes in the oEGM morphology (see Fig. 7B). As can be observed, the oEGM becomes progressively wider in comparison to the reference oEGM $o_{\textrm{ref}}(t)$ (Fig. 7B). Moreover, in addition to these morphological worsening, NLA and ORR worsened with increased interelectrode distances as well.

**Table 2:** *p* values derived from post-hoc tests comparing coefficients of configurations, stratified by metrics and distances

	NLA			ORR		
	${-}$	${-}$	${-}$	${-}$	${-}$	${-}$
1 mm	0.921	$<\,0.001$	$<\,0.01$	0.897	$<\,0.01$	$<\,0.001$
2 mm	0.391	$<\,0.05$	0.335	0.525	0.117	$<\,0.01$
3 mm	0.994	0.505	0.442	0.711	0.419	0.105
4 mm	0.933	0.491	0.712	0.899	0.905	0.664

	PW			MD		
	${-}$	${-}$	${-}$	${-}$	${-}$	${-}$
1 mm	0.605	0.911	0.361	0.536	0.689	0.147
2 mm	0.795	0.299	0.675	0.622	0.941	0.822
3 mm	0.380	0.266	0.971	0.463	0.999	0.464
4 mm	0.669	0.882	0.924	0.467	0.997	0.426

**Table 3:** *p* values derived from post-hoc tests comparing coefficients of distances, stratified by metrics and configurations

					and				and			
	1 mm	2 mm	3 mm	4 mm	1 mm	2 mm	3 mm	4 mm	1 mm	2 mm	3 mm	4 mm
NLA												
1 mm	1	–	–	–	1	–	–	–	1	–	–	–
2 mm	0.378	1	–	–	0.061	1	–	–	0.987	1	–	–
3 mm	$<\,0.05$	0.588	1	–	0.088	0.999	1	–	1	0.985	1	–
4 mm	$<\,0.05$	0.529	0.999	1	$<\,0.05$	0.979	0.946	1	0.999	0.995	0.999	1
ORR												
1 mm	1	–	–	–	1	–	–	–	1	–	–	–
2 mm	$<\,0.05$	1	–	–	0.066	1	–	–	0.256	1	–	–
3 mm	$<\,0.01$	0.962	1	–	$<\,0.01$	0.861	1	–	0.383	0.994	1	–
4 mm	$<\,0.01$	0.877	0.994	1	$<\,0.01$	0.505	0.928	1	0.737	0.84	0.938	1
PW												
1 mm	1	–	–	–	1	–	–	–	1	–	–	–
2 mm	0.159	1	–	–	0.956	1	–	–	0.73	1	–	–
3 mm	$<\,0.05$	0.791	1	–	0.876	0.995	1	–	0.239	0.827	1	–
4 mm	$<\,0.01$	0.464	0.95	1	0.297	0.594	0.741	1	$<\,0.01$	0.07	0.371	1
MD												
1 mm	1	–	–	–	1	–	–	–	1	–	–	–
2 mm	0.119	1	–	–	0.995	1	–	–	$<\,0.05$	1	–	–
3 mm	$<\,0.01$	0.725	1	–	0.76	0.882	1	–	$<\,0.001$	0.548	1	–
4 mm	$<\,0.05$	0.865	0.993	1	0.868	0.952	0.997	1	$<\,0.01$	0.639	0.999	1

## Discussion

Orientation-independent sensing (also referred as OIS) [31] for the estimation of bipolar electrograms is receiving great attention [23]. This technique overcomes the main limitation of bEGMs, which are highly dependent on bipole orientation, hence providing a low amplitude and fractionated signal when the wavefront arrives almost perpendicularly to the electrode pair [20]. Such a limitation is crucial, as low amplitude and identification of Complex Fractionated Atrial Electrograms (CFAEs) are key features in determining regions with anomalous conduction [32, 33].

Although claimed to be orientation-independent, it has been shown that some orientation dependency still applies [24]. Indeed, it has been proven that state-of-the-art oEGM reconstruction methods based on triangular cliques result in inaccurate estimations of the omnipole, even in perfectly homogeneous and plane propagation wavefronts [25]. As previously reported in a simulation study, oEGM estimation from the diagonal bEGMs of the clique corrects the temporal misalignments, hence improving oEGM estimation.

With this study, we aim to assess performance and limitations of OIS methods for oEGM estimation in a real scenario, employing a series of retrospective experiments with animal models. Several technical issues are tested, such as clique configuration and interelectrode distance. Parameters based on the form factor of the electric field loop and rejection to residual signal resulting from perpendicular electrode arrangement are considered. In addition, morphology distortion caused by increasing spacing between electrodes is analysed.

The fact that the stimulation electrode was placed in approximately the same location in all experiments forced a similar direction of the propagation wavefront, regardless of the experiment under study. As long as the accuracy of the electrical field loop reconstruction with the triangular clique strongly depends on the wavefront incidence angle, loop patterns were dissimilar for different triangle orientations. More specifically, equivalent loops were obtained from pairs of complementary triangles ( and ), whereas clearly different loops were obtained when comparing non-complementary triangles. This orientation-dependent property of the triangular clique, together with the specific arrangement of the experimental setting, caused one pair of complementary triangles to be consistently more accurate than the other. As a result, by using triangular cliques, correct oEGM estimations coexist with incorrect oEGM estimations. On the other hand, the cross-oriented configuration, being more robust to the wavefront direction, provided results as accurate as the best complementary triangle pair, and better in any case than the worst complementary triangle pair. This is an important benefit of the cross-oriented clique, as in clinical practice the wavefront can arrive randomly from any possible direction (even changing from activation to activation as the catheter moves).

According to our results, the aforementioned benefits of the cross-oriented configuration are obtained with close interelectrode spacing ($\le 2$ mm). In fact, it was found that increasing the interelectrode distance is another major performance limiting factor of oEGM estimation. For distances $\ge \,3$ mm, activation delays between neighbours become more noticeable, thus widening the bipolar pulse and changing its morphology waveform. Indeed, notched or fractionated pulses may be retrieved resulting from excessively delayed activations even in the case of a healthy cardiac tissue. In those cases, the cross-oriented configuration is negatively affected to a greater extent, as the interelectrode distance is scaled at a $\sqrt{2}$ factor with respect to triangular cliques. To tackle this hindrance, higher density catheters shall be designed. Moreover, by reducing interelectrode spacing, the resulting propagation wavefront would be better approximated by a planar and homogeneous wave within the dimensions of a clique. These findings are consistent with the recent work of Letchumy et al., which, aiming to characterise the effect of electrode number and interelectrode distance in the omnipole through an *in silico* set-up, concluded that 2 mm is an ideal interelectrode distance, and distances above 4 mm were less effective at characterising the underlying domain [34].

### Study limitations

Although experimental animal studies combine the control of experimental settings and the physiological behaviour, the isolated rabbit heart is certainly a limitation for the generalisation to human electrophysiological signals. The main question is to what extent the scaling factor of heart dimensions can affect the conclusions, especially those related to interelectrode distance. Nevertheless, it should be taken into account that conduction velocity of rabbit and human hearts are around the same order of magnitude. In our opinion, as long as the conduction velocity is more relevant than the heart size at local analyses, the conclusions may be extrapolated to a great extent.

Another limitation is that the stimulations were applied roughly in the same location for all experiments (obviously, with some random variations due to experimental settings and handling). Although this proved that the triangular clique arrangement failed consistently at certain angle orientations, it would have been interesting to test the robustness of the cross-orientation at all angles with an additional battery of experiments by placing the stimulation electrode at different sites. Nonetheless, this aspect is not sufficiently grounded to carry out a new series of experiments with animals. In any case, the cross-orientation was proven consistent along the complete experiment series as well as in previous simulation studies [25]. Future work shall be performed on endocavitary signals, given that the current has been performed on epicardial.

## Conclusions

In this paper, performance of orientation-independent sensing methods for cardiac signals are explored. For this, signals recorded from a high-density multielectrode during a retrospective experimental series of isolated perfused heart were employed. Electrical field loop reconstruction and orientation-independent bipolar activations for different technical configurations were compared. Several parameters based on loop shape, rejection ratio to the orthogonal residual signal and pulse morphology were defined. Our results concluded that interelectrode spacing not larger than 2 mm should be employed for accurate oEGM estimation. Moreover, if this condition is satisfied, a cross-orientation clique configuration is preferred over the triangular clique currently employed in clinical practice. This study opens a new standpoint on the reconstruction of oEGMs from high density multielectrode catheters, and a new vision towards the design of new devices and post-processing methods with improved features and performance.

## Supplementary Information

Below is the link to the electronic supplementary material.

Supplementary file 1 (pdf 973 KB)
