# Supplementary material for: Evaluation and assessment of clique arrangements for the estimation of omnipolar electrograms in high density electrode arrays: an experimental animal model study
Source: Phys Eng Sci Med. 2023 Jun 26;46(3):1193–204. doi: 10.1007/s13246-023-01287-8 (PMC10480253; doi:10.1007/s13246-023-01287-8)
Supplement: Supplementary file 1 — (pdf 973 KB) [file 13246_2023_1287_MOESM1_ESM.pdf]

## Supplementary Material 1. Statistical Analysis

DHARMa is an R package that aims to facilitate the problematic interpretation of residuals from generalised linear mixed models.

The package uses a simulation-based approach normalising the residuals to a scale 0-1. If the statistical model is correctly defined, its fitted residuals will all follow the same known distribution [35].

The R script lines used for the first model based on a random intercept dependence on the heart origin;

```
model <- lmer (log(metrics) ~ treatment * distance  +
               (1|rabbit) + (1|rabbit:treatment:distance),
               data=dat)
```

where `dat` is the dataframe containing the experimental data, *treatment* refers to the configuration ($C_{\triangle}, C_{\triangledown}$ and $C_{\times}$), `metrics` to the variable (NLA, ORR...), and `distance` to the interelectrode distance, describing `rabbit` the rabbit heart origin dependence.

The R script lines from the second model (the one used) built as a mixed model on the aggregated data (aggregation of subsamples of the rabbit heart origin);

```
aggregated_data <- aggregate (metrics ~ treatment +
                              distance + rabbit,
                              data = d, FUN= mean)
model_agg <- lmer(log(metrics) ~ distance * treatment +
                  (1|rabbit), data = aggregated_data)
```

A summary table from the regression models was retrieved, as well as the random effect variance of the heart origin, the p values were computed with Kenward-Roger Approximation for the degrees of freedom.

To calculate the randomised quantile residuals coming from the fitted model defined above and plot the DHARMa residual plot we made use of the following line of R code;

```
simulateResiduals(model_agg,plot=T)
```

Two plots were retrieved. A quantile-quantile plot containing added tests for correct distribution, dispersion and outliers, highly useful to detect overall deviations from the expected distribution.

A second figure was equally obtained with the DHARMa simulation, plotting the residuals against the rank transformed model predictions, including quantile regressions to discover trends.

A

| | log(NLA) | | |
|---|---|---|---|
| *Predictors* | *Estimates* | *CI* | *p* |
| (Intercept) | -2.15 | -2.51 – -1.79 | **<0.001** |
| distance [2mm] | 0.63 | 0.14 – 1.12 | **0.013** |
| distance [3mm] | 0.59 | 0.10 – 1.09 | **0.019** |
| distance [4mm] | 0.73 | 0.24 – 1.22 | **0.004** |
| treatment [$C_{\Delta}$] | 0.90 | 0.41 – 1.39 | **<0.001** |
| treatment [$C_X$] | -0.10 | -0.59 – 0.40 | 0.701 |
| distance [2mm] *treatment [$C_{\Delta}$] | -0.55 | -1.24 – 0.15 | 0.123 |
| distance [3mm] *treatment [$C_{\Delta}$] | -0.60 | -1.29 – 0.10 | 0.093 |
| distance [4mm] *treatment [$C_{\Delta}$] | -0.70 | -1.40 – -0.01 | **0.048** |
| distance [2mm] *treatment [$C_X$] | -0.23 | -0.93 – 0.47 | 0.513 |
| distance [3mm] *treatment [$C_X$] | 0.12 | -0.58 – 0.82 | 0.730 |
| distance [4mm] *treatment [$C_X$] | 0.01 | -0.69 – 0.70 | 0.983 |
| **Random Effects** | | | |
| $\sigma^2$ | 0.28 | | |
| $\tau_{00\ rabbit}$ | 0.02 | | |
| ICC | 0.06 | | |
| $N_{rabbit}$ | 9 | | |
| Observations | 108 | | |
| Marginal $R^2$ / Conditional $R^2$ | 0.282 / 0.325 | | |

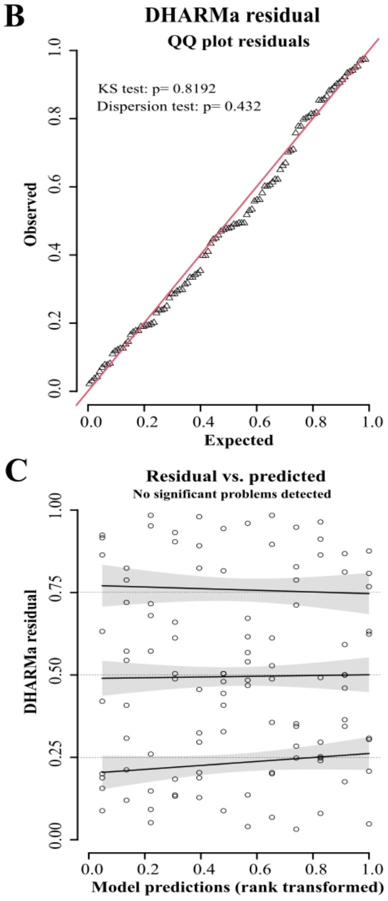


Figure 8: Residual diagnostics for Hierarchical regression model in NLA metric. A. Regression Model table; B. Residuals QQ Plot; C. Residuals plot over their expected value.

**A**

| | log(ORR) | | |
|---|---|---|---|
| *Predictors* | *Estimates* | *CI* | *p* |
| (Intercept) | 1.63 | 1.38 – 1.87 | **<0.001** |
| distance [2mm] | -0.39 | -0.70 – -0.08 | **0.014** |
| distance [3mm] | -0.51 | -0.82 – -0.20 | **0.001** |
| distance [4mm] | -0.60 | -0.91 – -0.30 | **<0.001** |
| treatment [$C_{\Delta}$] | -0.58 | -0.89 – -0.27 | **<0.001** |
| treatment [$C_X$] | -0.07 | -0.38 – 0.24 | 0.658 |
| distance [2mm] *treatment [$C_{\Delta}$] | 0.10 | -0.33 – 0.54 | 0.646 |
| distance [3mm] *treatment [$C_{\Delta}$] | 0.26 | -0.17 – 0.70 | 0.236 |
| distance [4mm] *treatment [$C_{\Delta}$] | 0.45 | 0.01 – 0.88 | **0.045** |
| distance [2mm] *treatment [$C_X$] | -0.10 | -0.54 – 0.34 | 0.651 |
| distance [3mm] *treatment [$C_X$] | -0.05 | -0.49 – 0.38 | 0.808 |
| distance [4mm] *treatment [$C_X$] | 0.00 | -0.43 – 0.44 | 0.996 |
| **Random Effects** | | | |
| $\sigma^2$ | 0.11 | | |
| $\tau_{00\ rabbit}$ | 0.03 | | |
| ICC | 0.22 | | |
| $N_{rabbit}$ | 9 | | |
| Observations | 108 | | |
| Marginal $R^2$ / Conditional $R^2$ | 0.326 / 0.476 | | |

**B**

**C**

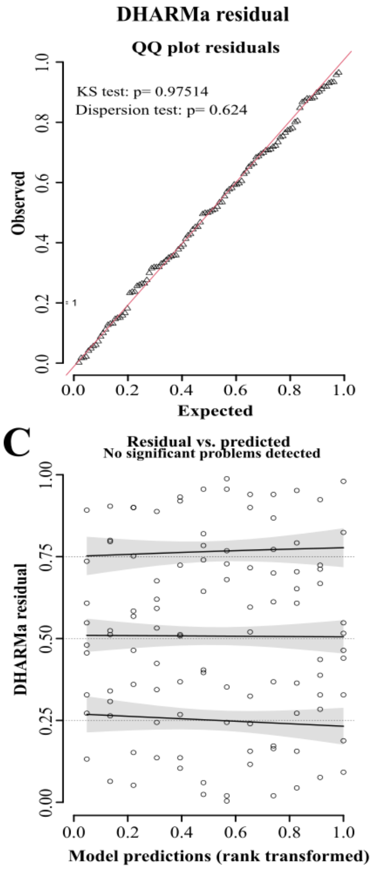


Figure 9: Residual diagnostics for Hierarchical regression model in ORR metric; A. Regression Model table; B. Residuals QQ Plot; C. Residuals over model predictions.

**A**

| | | log(PW) | |
|---|---|---|---|
| *Predictors* | *Estimates* | *CI* | *p* |
| (Intercept) | 3.84 | 3.47 – 4.22 | **<0.001** |
| distance [2mm] | 0.07 | -0.20 – 0.33 | 0.610 |
| distance [3mm] | 0.10 | -0.17 – 0.37 | 0.454 |
| distance [4mm] | 0.24 | -0.03 – 0.50 | 0.081 |
| treatment [$C_{\Delta}$] | -0.18 | -0.45 – 0.08 | 0.174 |
| treatment [$C_X$] | -0.13 | -0.39 – 0.14 | 0.340 |
| distance [2mm] * treatment [$C_{\Delta}$] | 0.07 | -0.31 – 0.45 | 0.712 |
| distance [3mm] * treatment [$C_{\Delta}$] | 0.15 | -0.22 – 0.53 | 0.422 |
| distance [4mm] * treatment [$C_{\Delta}$] | 0.23 | -0.14 – 0.61 | 0.219 |
| distance [2mm] * treatment [$C_X$] | 0.21 | -0.16 – 0.59 | 0.259 |
| distance [3mm] * treatment [$C_X$] | 0.31 | -0.07 – 0.68 | 0.108 |
| distance [4mm] * treatment [$C_X$] | 0.24 | -0.13 – 0.62 | 0.203 |
| **Random Effects** | | | |
| $\sigma^2$ | 0.08 | | |
| $\tau_{00\ rabbit}$ | 0.24 | | |
| ICC | 0.75 | | |
| $N_{rabbit}$ | 9 | | |
| Observations | 108 | | |
| Marginal $R^2$ / Conditional $R^2$ | 0.077 / 0.768 | | |

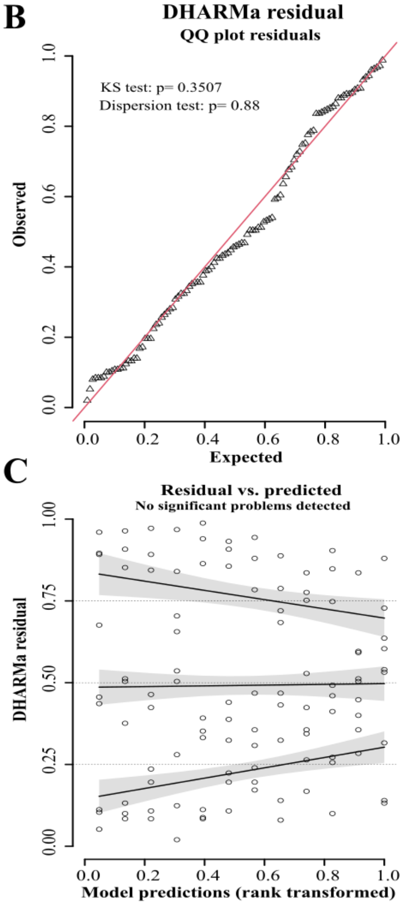


Figure 10: Residual diagnostics for Hierarchical regression model in PW metric; A. Regression Model table; B. Residuals QQ Plot; C. Residuals over model predictions.

**A**

| | | log(MD) | |
|---|---|---|---|
| *Predictors* | *Estimates* | *CI* | *p* |
| (Intercept) | -2.26 | -2.47 – -2.05 | **<0.001** |
| distance [2mm] | 0.03 | -0.21 – 0.27 | 0.808 |
| distance [3mm] | 0.12 | -0.12 – 0.36 | 0.329 |
| distance [4mm] | 0.09 | -0.15 – 0.33 | 0.443 |
| treatment [$C_{\Delta}$] | -0.23 | -0.47 – 0.01 | 0.061 |
| treatment [$C_X$] | -0.13 | -0.37 – 0.11 | 0.288 |
| distance [2mm] * treatment [$C_{\Delta}$] | 0.30 | -0.04 – 0.64 | 0.082 |
| distance [3mm] * treatment [$C_{\Delta}$] | 0.37 | 0.03 – 0.71 | **0.032** |
| distance [4mm] * treatment [$C_{\Delta}$] | 0.38 | 0.04 – 0.72 | **0.029** |
| distance [2mm] * treatment [$C_X$] | 0.24 | -0.10 – 0.58 | 0.161 |
| distance [3mm] * treatment [$C_X$] | 0.28 | -0.06 – 0.62 | 0.107 |
| distance [4mm] * treatment [$C_X$] | 0.27 | -0.07 – 0.61 | 0.115 |
| **Random Effects** | | | |
| $\sigma^2$ | 0.07 | | |
| $\tau_{00\ rabbit}$ | 0.03 | | |
| ICC | 0.34 | | |
| $N_{rabbit}$ | 9 | | |
| Observations | 108 | | |
| Marginal $R^2$ / Conditional $R^2$ | 0.187 / 0.460 | | |

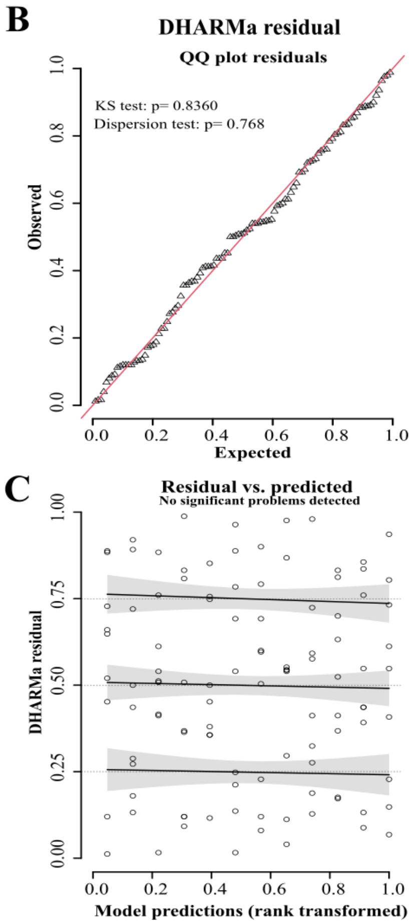


Figure 11: Residual diagnostics for Hierarchical regression model in MD metric; A. Regression Model table; B. Residuals QQ Plot; C. Residuals over model predictions.

## Supplementary Material 2. Code

The full script will be provided in a *github* link when the manuscript is accepted for publication.
